# Cognitive Predictors of Adaptive Behaviour in Children With Down Syndrome: A Systematic Review

**DOI:** 10.1111/jar.70201

**Published:** 2026-02-17

**Authors:** Fernanda Silva Pereira, Renata Maria Silva Santos, Luiz Humberto Souza Junior, Núbia Hadassa França Ferreira de Carvalho, Clara de Paula Gomes, Alice Martins Ferreira, Marco Aurélio Romano‐Silva, Leandro Fernandes Malloy‐Diniz, Débora Marques de Miranda

**Affiliations:** ^1^ Graduate Program in Child and Adolescent Health in Faculty of Medicine Federal University of Minas Gerais (UFMG) Belo Horizonte Brazil; ^2^ Graduate Program in Molecular Medicine, Faculty of Medicine Federal University of Minas Gerais (UFMG) Belo Horizonte Brazil; ^3^ Department of Psychiatry, Faculty of Medicine Federal University of Minas Gerais (UFMG) Belo Horizonte Brazil; ^4^ Department of Pediatrics, Faculty of Medicine Federal University of Minas Gerais (UFMG) Belo Horizonte Brazil

**Keywords:** adaptive behaviour, children, cognitive skills, down syndrome, executive function, systematic review, trisomy 21

## Abstract

**Background:**

Children with Down Syndrome, caused by trisomy 21, frequently exhibit deficits in adaptive behaviour. This systematic review aimed to identify predictors of adaptive behaviour in children with Down Syndrome.

**Method:**

The review followed PRISMA guidelines and was registered in PROSPERO (CRD420251071028). Searches were conducted in June 2025 in PsycINFO, Scopus, ERIC, Web of Science and PubMed.

**Results:**

A total of 257 articles were yielded, of which eight met the eligibility criteria. Across studies, executive functions consistently stood out as the strongest predictors of adaptive behaviour, explaining variance in communication, socialisation and daily living skills. General cognitive skills were also associated, although with smaller effects.

**Conclusions:**

Overall, these findings highlight that deficits in executive and intellectual functioning constrain autonomy and everyday functioning in Down Syndrome, underscoring the importance of targeted interventions to improve adaptive outcomes.

## 1 Introduction

Down Syndrome is a congenital condition with a genetic basis characterised by trisomy of chromosome 21, with an estimated incidence of approximately 1 in 800 live births worldwide, although this number varies across different regions (Laignier et al. 2021; Corona‐Rivera et al. 2019). The clinical profile of Down Syndrome includes craniofacial alterations (Roizen and Patterson 2003), hypotonia, and an increased risk of cardiovascular conditions (Bull 2020), in addition to impairments in multiple domains of development, particularly cognitive skills and adaptive behaviour (Crombie and Gunn 1998; Lukowski et al. 2019).

From a conceptual viewpoint, adaptive behaviour corresponds to a complex and multifactorial construct, composed of an integrated set of conceptual, social and practical skills that support the child's progressive autonomy and functional participation in daily and community contexts (APA 2022; Sparrow et al. 2016). These skills develop from the dynamic interaction between cognitive resources and environmental conditions (Schalock et al. 2010) and encompass domains such as language, socialisation and independence in daily living activities, which are expressed in functional capacities related to autonomy from caregivers, school performance and quality of life (Tassé et al. 2016). Higher levels of adaptive behaviour are associated with greater independence in adulthood, better academic performance and lower frequency of maladaptive behaviours (Bornstein et al. 2013; Racz et al. 2017; Woolf et al. 2010).

Longitudinal evidence indicates that, in children with Down syndrome, the acquisition of adaptive behaviour milestones follows a similar sequence to that observed in typically developing children, but with a slower pace and a considerably lower functional plateau (Van Duijn et al. 2010). Compared to typical development, a global delay in adaptive behaviour is observed from the first year of life, with variations in the adaptive behaviour throughout development, such that young children tend to show strengths in socialisation and weaknesses in communication, while adolescents around 12 years old exhibit a more homogeneous profile (Van Duijn et al. 2010).

Parallel to adaptive limitations, cognitive skills in individuals with Down syndrome also show wide heterogeneity. Cognitive functions correspond to the basic mental processes responsible for perceiving, analyzing, storing and utilizing information (Neisser 1967). Among these processes, executive functions stand out, traditionally described by three central components: working memory, inhibitory control and cognitive flexibility (Diamond 2013; Lehto et al. 2003), which play a fundamental role in academic success (Borella et al. 2010; Duncan et al. 2007), quality of life (Davis et al. 2010) and mental health (Lui and Tannock 2007).

In addition to executive functions, other cognitive skills also deserve emphasis, such as visual perception, defined as the ability to integrate and mentally construct fragments of visual information for recognition or functional use (Wuang et al. 2010). Difficulties in this domain can significantly interfere with academic learning, participation in school activities, and the demands of daily life. Furthermore, recurrent experiences of failure associated with these difficulties can negatively affect the psychosocial development of these children (Vicari et al. 2005; Wan et al. 2015).

Complementarily, the literature indicates that intelligence quotient (IQ) is one of the most investigated and most heterogeneous constructs in Down syndrome, generally falling within the range associated with intellectual disability (between 40 and 70 points), although wide individual variability exists. This heterogeneity, combined with the limited sensitivity of available instruments, represents a significant methodological challenge in evaluating intelligence in this population. Moreover, the slower pace of cognitive development means that comparisons based solely on chronological age result in an apparent decline in IQ scores over time, even in the presence of real gains in skills (Channell et al. 2021).

Considering the complexity and multifactorial nature of adaptive behaviour, as well as the heterogeneity of cognitive profiles described in children with Down syndrome, evidence suggests that alterations in different cognitive domains are associated with impairments in adaptive behaviour throughout development (Daunhauer et al. 2014; Tomaszewski et al. 2018). Although the relevance of cognition as a predictor of adaptive behaviour is widely recognised, the literature still presents important gaps regarding the systematic investigation of this relationship specifically in childhood, a period marked by greater developmental variability (Hendrix et al. 2021). In this sense, an integrated understanding of the development of cognitive skills and adaptive behaviour in this population can contribute to the improvement of therapeutic support and the formulation of more appropriate educational strategies.

In this context, identifying the cognitive skills most directly associated with adaptive behaviour is fundamental to inform the planning of targeted interventions, capable of promoting the autonomy, inclusion and social participation of children with Down syndrome in clinical and educational settings. Considering that adaptive behaviour constitutes a central component of independence and personal development, and represents a relevant area of limitation in this population, a systematisation of the available evidence becomes necessary. Thus, this systematic review aimed to examine which cognitive skills predicted adaptive behaviour in children with Down syndrome.

## 2 Method

This review was conducted in compliance with the Preferred Reporting Items for Systematic Reviews and Meta‐Analyses (PRISMA), a protocol that standardises evidence collection (Page et al. 2021), and was registered in the International Prospective Register of Systematic Reviews (PROSPERO) under number CRD420251071028. A search was carried out between June 6 and June 9, 2025, guided by the following research question: *What cognitive factors predicted adaptive behaviour among children with Down syndrome?* The searches were conducted using the ‘All Fields’ option by default across databases; however, for PsycINFO, the search was restricted to the Title field due to excessive retrieval of records outside the scope of this review. The complete search syntaxes used for each database are provided in Table S1.

Following the PECO framework, the population of interest comprised children with the diagnosis of Down Syndrome. Studies with or without a control group were considered eligible. The outcomes included measures of adaptive behaviour, encompassing daily living skills and social skills, as well as deficits in adaptive behaviour.

### 2.1 Inclusion Criteria

- Studies with children diagnosed with Down Syndrome (trisomy 21).
- Aged 0–12 years, or with a mean age up to 12 years. Studies with participants older than 12 years were included only if they reported stratified data for a subgroup of participants aged 0–12 years, which allowed for the extraction of the relevant sub‐data concerning the target population.
- In English.
- Original empirical studies (quantitative, observational or experimental).
- Studies investigating the relationship between cognitive skills (e.g., executive function, memory, general cognition) and adaptive behaviour (e.g., daily living skills, social skills).

### 2.2 Exclusion Criteria

- Individuals presenting neuropsychiatric comorbidities, such as Autism Spectrum Disorder (ASD) or Attention‐Deficit/Hyperactivity Disorder (ADHD), due to their well‐established impact on cognitive functions and adaptive behaviour.
- Studies employing non‐standardised instruments to assess cognitive skills and relevant outcomes.
- Qualitative studies.
- Case series or reports, literature reviews, grey literature, editorials and abstracts from scientific conferences.

### 2.3 Screening Procedure

The screening process was conducted in pairs and in a blinded manner. After removing duplicates, two independent reviewers selected articles based on title and abstract, according to the inclusion and exclusion criteria. Each reviewer independently identified articles eligible for full‐text review. Studies that generated conflicts had their inclusion or exclusion determined by consensus with a third reviewer.

### 2.4 Data Extraction and Data Synthesis

Data were extracted using a standardised form that included the following information: first author, year of publication, country, study design, sample characteristics, study objectives, instruments used to assess cognitive skills and the outcomes examined in each study, and the main findings. Effect size measures will be harmonised whenever possible to improve comparability across studies. Specifically, values reported as eta‐squared (*η* ^2^), the proportion of explained variance (*R* ^2^, including adjusted *R* ^2^), t statistics, Cohen's d, and chi‐square (*χ* ^2^, 2 × 2 tables) will be converted into correlation coefficients (*r*) when appropriate. Standardised regression coefficients (*β*) will be retained and interpreted according to conventional benchmarks for effect size magnitude (small ≈0.10; moderate ≈0.30; large ≈0.50) (Cohen 1988; Borenstein et al. 2009).

### 2.5 Quality Assessment

The quality assessment of the included articles was conducted using the Joanna Briggs Institute (JBI) Critical Appraisal Tool. Articles were independently evaluated by two reviewers. Studies rated as high quality were expected to clearly describe the method used to assess cognitive skills and adaptive behaviour, as well as to address the identification of confounding factors that could impact study outcomes. Furthermore, studies were expected to present results measured in a valid and reliable manner, with appropriate statistical analyses.

Although the JBI guidelines do not specify cut‐off points for classifying studies as ‘Good’, ‘Fair’ or ‘Low’ quality, this review prioritised, based on consensus among the review team, the items most relevant to the main research question. Each item of the JBI critical appraisal checklists was rated as ‘Yes’, ‘No’, ‘Unclear’ or ‘Not applicable’, in accordance with JBI guidelines. Items rated as ‘Not applicable’ were excluded from the total score to avoid distortion of the overall quality assessment. For both study designs, methodological quality was classified based on the percentage of applicable items rated as ‘Yes’. Studies were categorised as good quality when ≥ 70% of applicable items were met, fair quality when 50%–69% were met, and low quality when < 50% of applicable items were met. These criteria were applied consistently across all included studies. Accordingly, for cross‐sectional studies, attention was focused on items 3, 4, 5, 6, 7 and 8. For cohort studies, the most important items were questions 2, 3, 4, 5, 7, 8, 9, 10 and 11. Thus, studies considered of ‘good’ quality were required to answer the selected questions affirmatively (‘Yes’).

## 3 Results

**FIGURE 1:**
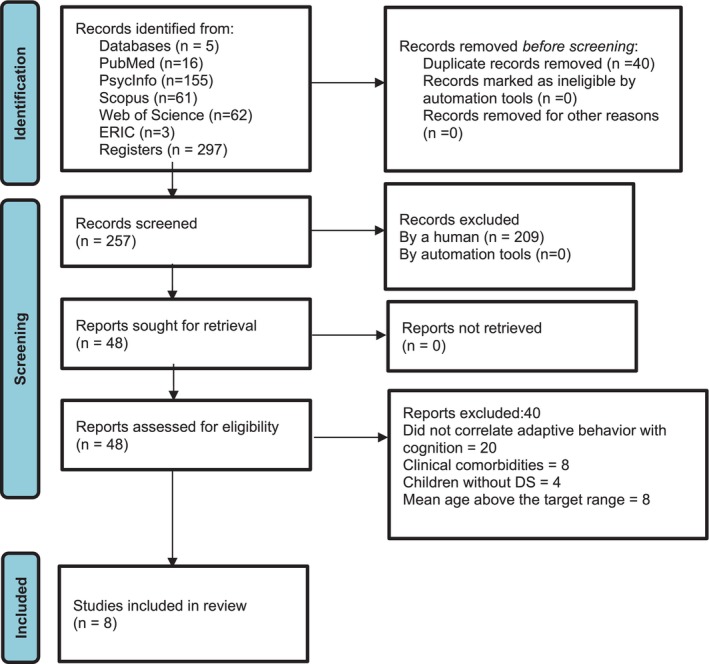
PRISMA flow diagram.

Of the 297 studies retrieved from the databases, duplicates and studies meeting the exclusion criteria were removed, resulting in eight articles included in the review. The full study selection process is detailed according to the PRISMA protocol in Figure 1.

### 3.1 Study Characteristics

Among the included studies, six employed a cross‐sectional design and two were longitudinal, published between 1998 and 2024, encompassing 683 participants (54% male) with a mean age of 8.14 years (SD = 3.32). Across studies, participant ages ranged broadly from early preschool to adolescence (approximately 4.7–16 years), capturing multiple developmental stages. Four studies were conducted in the United States, and one each in Australia, Israel, China and Italy. One study additionally included a typically developing comparison group; however, only data from participants with Down syndrome were considered in the present synthesis. Half of the studies examined executive functions, which were found to correlate with subdomains of adaptive behaviour. The most commonly used instruments for assessing cognitive function were the Stanford–Binet and the Behaviour Rating Inventory of Executive Function (BRIEF), while adaptive behaviour was primarily measured using the Vineland Adaptive Behaviour Scales. Table 1 provides a comprehensive overview of the study characteristics and main associations, with studies arranged in ascending chronological order to facilitate the analysis of temporal trends.

**TABLE 1:** Descriptive characteristics of the included studies.

Author/year/country/type of study	*N*|age|gender %|purpose	Cognitive assessement	Adaptive assessment	Major associations
Crombie and Gunn (1998) Australia Cohort	*N:* Cohort 1: 33 children Cohort 2: 41 children Mean age: 12.5 years Gender: 45 (60.8%) boys To investigate the long‐term effects of early intervention on the development of adolescents with Down Syndrome.	Stanford‐Binet Intelligence Scale (Form L‐M); Hiskey Nebraska Test of Learning Aptitude (HNTLA)	AAMD Adaptive Behaviour Scale—School Edition (ABS–SE)	Verbal cognition was positively associated with community self‐sufficiency, with a large effect size (*r* = 0.76, *p* < 0.00).
Rihtman et al. (2010) Cross‐sectional Israel	*N*: 60 Mean age: 9.2 years Gender: 33 (55%) boys To investigate the relationship between cognitive skills and adaptive behaviour (functional participation) in children with Down Syndrome aged 6–16 years who received early intervention.	Stanford‐Binet Intelligence Scale, 4th Edition (SBIS); Beery‐Buktenica Developmental Test of Visual‐Motor Integration, 5th Edition (Beery VMI).	Vineland 3	Visuomotor integration and visual skills showed moderate effects with adaptive behaviour (*r* = 0.38) and (*r* = 0.24), respectively.
Wuang and Su (2011) China Cross‐sectional	*N*: 206 Mean age: 8 years and 1 month Gender: 105 (50.9%) girls To investigate the developmental profile of sensory processing and visual organisation skills, body functions classified according to the WHO ICF model, and their impact in children with Down Syndrome.	Hooper Visual Organisation Test; Sensory Profile; Wechsler Intelligence Scale for Children—Third Edition (WISC‐III); School Function Assessment—Chinese Version (SFA‐C).	Vineland Adaptive Behaviour Scale—Chinese Version (VABS‐C)	Visual organisation demonstrated moderate effects across adaptive behaviour domains, including communication (*r* = 0.38), daily living skills (*r* = 0.40), motor skills (*r* = 0.47), and the overall adaptive behaviour score (*r* = 0.45).
Will et al. (2016) United States Cross‐sectional	*N*: 24 Mean age: 6 years and 4 months Gender: 13 (54%) boys This study characterised maladaptive behaviour in school‐aged children with Down Syndrome and examined the extent to which maladaptive behaviours are associated with school function.	Leiter‐R Brief (QI)	BASC 2 School Function Assessment (SFA)	Attention problems significantly predicted performance in task completion, a practical adaptive behaviour, accounting for 44% of the variance (adjusted *R* ^2^ = 0.44; *b* = −2.24; *p* = 0.00). The multivariate analysis confirmed the overall effect (*F*(2,16) = 6.24; *p* = 0.01)
Daunhauer et al. (2017) United States Transversal	*N*: 80 (42 with SD and 38 typically developing TD) Mean Age: 7 years 9 months (SD); 3 years 4 months (TD) Gender: 26 (62%) boys (SD); 16 (42.1%) boys (TD) To investigate the executive function performance profile of school‐aged children with SD, using both performance‐based measures and caregiver‐report (ratings‐based) measures.	Behaviour Rating Inventory of Executive Function (BRIEF); Digit Span Test (WISC‐IV); Inhibitory Pathway Task (IPT); Cognitive Flexibility Sorting Task (CFS‐Sort); Corsi Block‐Tapping Task—Backward.	Adaptive Behaviour/Self‐care‐ Paediatric Evaluation of Disability Inventory	Difficulties in working memory showed a moderate negative effect on adaptive behaviour (*β* = −0.37; *p* = 0.03), with the model explaining 24% of the variance in this outcome (adjusted *R* ^2^ = 0.24; *p* = 0.01).
Will et al. (2021) United States Transversal	*N*: 68 Mean Age: 12.56 years Gender: 36 (53.6%) boys To characterise the relationship between parent‐reported executive functions and parent‐reported adaptive behaviours, and to identify which aspects of executive functions are the most salient predictors of adaptive behaviour.	BRIEF‐2	Vineland 3	Difficulties in working memory were the most robust predictor of adaptive behaviour, showing a large effect on communication (*β* = −1.06; *p* = 0.00; *η* ^2^ = 0.14) and socialisation (*β* = −0.94; *p* = 0.00; *η* ^2^ = 0.17), and a moderate effect on daily living skills (*β* = −0.69; *p* = 0.03; *η* ^2^ = 0.07). Difficulties in cognitive flexibility showed a moderate effect on daily living skills (*β* = −0.41; *p* = 0.02; *η* ^2^ = 0.08) and socialisation (*β* = −0.41; *p* = 0.01; *η* ^2^ = 0.10).
Onnivello et al. (2022) Italy Cross‐sectional	*N*: 100 (40 preschoolers; 60 school‐aged) Mean Age: 4.7 years (preschoolers) and 11.7 years (school‐aged) Gender: 25 (62.5%) boys; 38 (63.3%) boys To investigate how different components of executive functions are associated with adaptive behaviour in adolescents with SD, focusing on which executive domains best predict adaptive behaviour.	BRIEF 2 e BRIEF‐P	Vineland II	Difficulties in behavioural regulation (inhibition, cognitive flexibility, and emotional control) showed a strong negative correlation with social skills (*r* = −0.52; *p* < 0.01) and a moderate correlation with communication (*r* = −0.44; *p* < 0.01). Difficulties in working memory were moderately correlated with community use (*r* = −0.48; *p* < 0.01), functional academic skills (*r* = −0.43; *p* < 0.05), and health and safety (*r* = −0.41; *p* < 0.05). Deficits in planning were also moderately negative correlated with overall practical skills (*r* = −0.45; *p* < 0.01) and self‐care (*r* = −0.39; *p* < 0.05). Finally, specific difficulties in inhibition control showed moderate correlations with leisure (*r* = −0.42; *p* < 0.05) and community use (*r* = −0.37; *p* < 0.05).
Van Deusen et al. (2024) United States Longitudinal	*N*: 71 Mean Age: 5.23 years Gender: 36 (50.7%) boys To examine the simultaneous and longitudinal association between performance in daily living skills (DLS) and a set of cognitive skills, specifically executive function (EF), that are thought to support the acquisition of DLS.	Bayley Scales of Infant and Toddler Development—4th Edition (Bayley‐4) Working Memory: Garage Game Task Inhibitory Control: Snack Delay Task Cognitive Flexibility: Adapted Reverse Categorisation Task Stanford–Binet Intelligence Scales—5th Edition: Abbreviated IQ	Vineland 3	Executive functions significantly predicted daily living skills, explaining 21%–37% of the variance (*R* ^2^ = 0.21–0.37), representing a moderate effect. Inhibitory control showed a moderate‐to‐strong and highly significant association (*β* = 0.40–0.56; *p* < 0.00), whereas cognitive flexibility demonstrated a low‐to‐moderate association with borderline significance (*β* = 0.28; *p* = 0.04).

Abbreviations: AAMD, American Association on Mental Deficiency; BASC‐2, Behaviour Assessment System for Children—Second Edition; BRIEF 2, school‐age children; BRIEF‐ P, Behaviour Rating Inventory of Executive Function Preschool; BRIEF, Behaviour Rating Inventory of Executive Function; CFS‐Sort, Cognitive Flexibility Sorting Task.

Overall, adaptive behaviour was primarily assessed using the Vineland Adaptive Behaviour Scales (Second and Third Editions; VABS‐II and VABS‐III), followed by the Adaptive Behaviour Scale—School Edition (ABS‐SE), the Behaviour Assessment System for Children, Second Edition (BASC‐II), the Paediatric Evaluation of Disability Inventory (PEDI), and the School Function Assessment (SFA).

Across instruments, the most frequently assessed domains were Daily Living Skills/Practical skills and Socialisation, followed by Communication. The VABS‐II/III, the most commonly used instrument, evaluates adaptive behaviour across the domains of Communication, Daily Living Skills, Socialisation and Motor Skills, with an optional Maladaptive Behaviour Index. The ABS‐SE assesses adaptive behaviour through domains related to personal and community self‐sufficiency, personal and social responsibility and social adaptation. The BASC‐II includes adaptive behaviour composites alongside internalising and externalising symptoms, based on parent‐ and teacher‐report. The PEDI focuses primarily on Daily Living Skills, Mobility, Social/Cognitive functioning and Responsibility, while the SFA evaluates functional participation and performance in the school context.

Taken together, domains related to daily living/practical skills and social functioning emerged as the most frequently used adaptive behaviour outcomes across the included studies.

The results of this review indicate that adaptive behaviour in Down syndrome is a multidimensional construct supported by different cognitive domains, with emphasis on executive functions (EF). Working memory and inhibitory control emerge as the most consistent and robust predictors, showing effects that vary from moderate to large in critical domains such as communication, daily living skills and socialisation. Additionally, cognitive flexibility and planning demonstrated a direct impact on practical autonomy and behavioural self‐regulation, although with statistical variability across studies. Beyond the EFs, visuospatial organisation and global intelligence indicators (IQ) complement this profile, explaining significant portions of the variance in community and motor self‐sufficiency competencies. Collectively, these pieces of evidence suggest that the functional performance of children with DS does not depend on an isolated ability, but on an integrated cognitive architecture, where specific executive deficits act as primary barriers to adaptation to the social and school environment.

### 3.2 Main Associations

To ensure consistency in the synthesis, statistical conversions were conducted whenever possible to produce more homogeneous results. *P*‐values were retained when originally reported; when absent, effect sizes are presented alone to convey the strength of the associations. In each outcome block, the magnitudes of effect for the reported associations are summarised to facilitate overall comparability across studies.

#### 3.2.1 Cognitive Flexibility and Adaptive Behaviour

Within the scope of executive components, we begin the analysis by exploring how the capacity for mental shifting influences functional adjustment. Below, we synthesise the associations identified between cognitive flexibility and adaptive behaviour. In populations of children with Down syndrome, deficits in cognitive flexibility were negatively correlated with daily living skills (*β* = −0.41; *p* = 0.023; *η* ^2^ = 0.08 → *r* ≈0.28) and with socialisation (*β* = −0.41; *p* = 0.011; *η* ^2^ = 0.10 → *r* ≈0.32) (Will et al. 2021). In another study, cognitive flexibility was identified as a predictor of daily living skills (*β* = 0.28; *p* = 0.04), while inhibitory control emerged as a stronger predictor (*β* = 0.40–0.56; *p* < 0.001) (Van Deusen et al. 2024). Difficulties in behavioural regulation, including flexibility, inhibition and emotional control, were negatively correlated with social skills (*r* = −0.52; *p* < 0.01) and communication (*r* = −0.44; *p* < 0.01) (Onnivello et al. 2022). Overall, most associations between cognitive flexibility and adaptive behaviour were of small to moderate magnitude, whereas inhibitory control demonstrated large effects on daily living skills.

#### 3.2.2 Inhibitory Control and Adaptive Behaviour

Beyond flexibility, the literature highlights the regulatory role of suppressing impulsive responses. This subsection details the influence of inhibitory control on daily living and socialisation skills. Performance in inhibitory control showed a positive and significant correlation with daily living skills (*β* = 0.40–0.56; *p* < 0.001) (Van Deusen et al. 2024). Difficulties in inhibitory control were also negatively correlated with leisure activities (*r* = −0.42; *p* < 0.05) and community use (*r* = −0.37; *p* < 0.05), both relevant components of adaptive behaviour (Onnivello et al. 2022). Only one study did not find a significant association between inhibitory control and adaptive behaviour and a specific subdomain of adaptive behaviour (self‐care) (Daunhauer et al. 2017). Overall, associations between inhibitory control and adaptive behaviour were of moderate to large magnitude, with the strongest effects observed for daily living skills.

#### 3.2.3 Working Memory and Adaptive Behaviour

Considering the need for retention and manipulation of information for the execution of daily tasks, we present below the evidence on the relationship between working memory and adaptive performance. Difficulties in working memory consistently predicted poorer adaptive behaviour in children with Down syndrome. Will et al. (2021) reported significant associations across all three domains of adaptive behaviour: communication (*β* = −1.06; *η* ^2^ = 0.14, large effect), daily living skills (*β* = −0.69; *η* ^2^ = 0.07, moderate effect) and socialisation (*β* = −0.94; *η* ^2^ = 0.17, large effect). Daunhauer et al. (2017) found that parent‐reported working memory difficulties significantly predicted self‐care performance (*β* = −0.37), with the model explaining 24% of the variance (adjusted *R* ^2^ = 0.24; approximated *r* ≈0.49, large effect). Onnivello et al. (2022) observed moderate negative correlations between working memory difficulties and community use (*r* = −0.48), functional academic skills (*r* = −0.43) and health and safety (*r* = −0.41). Working memory impairments were therefore associated with moderate‐to‐large reductions in adaptive behaviour.

#### 3.2.4 Planning and Adaptive Behaviour

The execution of complex goals requires higher‐order functions that organise action. In this section, we describe how planning difficulties directly impact the practical competencies of children with Down Syndrome. Planning, a higher‐order executive function, showed significant associations with adaptive behaviour in children with Down syndrome. Onnivello et al. (2022) reported negative correlations between planning difficulties and overall practical skills (*r* = −0.45) and self‐care (*r* = −0.39), indicating a direct impact on daily activities. Daunhauer et al. (2017) found that planning was not a statistically significant predictor of adaptive behaviour when reported by parents (*β* = −0.29, *p* = 0.098). Planning difficulties were therefore associated with small‐to‐moderate reductions in adaptive behaviour.

#### 3.2.5 Visual Organisation and Adaptive Behaviour

Beyond the exclusive analysis of executive functions, it is imperative to consider sensory and motor support. Below, we summarise the correlations between visuospatial cognition and daily life domains. Visuospatial cognition, described as visual organisation and visuomotor integration, showed significant associations with adaptive domains in children with Down syndrome. Wuang and Su (2011) reported positive correlations between visual organisation and communication (*r* = 0.38), daily living skills (*r* = 0.40), socialisation (*r* = 0.34), motor skills (*r* = 0.47) and the composite adaptive score (*r* = 0.45). Rihtman et al. (2010) found that visuomotor integration was positively correlated with overall adaptive behaviour (*r* = 0.38), and visual skills showed a smaller positive association (*r* = 0.24). Visual‐perceptual and visuomotor abilities were therefore associated with small‐to‐moderate improvements in adaptive behaviour.

#### 3.2.6 IQ, Verbal and Nonverbal Cognition, and Adaptive Behaviour

Finally, we examine the impact of global developmental indicators, addressing how IQ and verbal and non‐verbal skills contribute to the variance in self‐sufficiency indices. Global cognitive indicators, including intelligence quotient (IQ), verbal cognition and nonverbal cognition, showed associations with adaptive behaviour in children with Down syndrome. Crombie and Gunn (1998) reported a strong correlation between verbal cognition and community self‐sufficiency (*r* = 0.76) and a moderate correlation for nonverbal cognition (*r* = 0.53). Regression analyses indicated that nonverbal cognition accounted for substantial variance in adaptive domains: personal self‐sufficiency (24.5%, adjusted *R* ^2^ = 0.245 → *r* ≈0.50, large effect), community self‐sufficiency (65.2%, adjusted *R* ^2^ = 0.652 → *r* ≈0.81, large effect), and personal‐social responsibility (31.3%, adjusted *R* ^2^ = 0.313 → *r* ≈0.56, large effect). Will et al. (2016) found that attentional difficulties significantly predicted adaptive behaviour in school. Impaired attention was associated with challenges in task engagement and completion, a component of practical adaptive behaviour. Regression analyses showed that attention problems explained 44% of the variance in task completion (*b* = −2.24; adjusted *R* ^2^ = 0.44 → *r* ≈0.66, large effect), and the multivariate model confirmed the overall effect (*F*(2,16) = 6.24; *p* = 0.010). Cognitive abilities were therefore associated with moderate‐to‐large variations in adaptive behaviour across multiple domains.

### 3.3 Methodological Quality Assessment

Most of the included studies were rated as fair quality (5/8, 62.5%), while three studies (3/8, 37.5%) were classified as good quality; no study was rated as low quality. The detailed results of the quality assessment are presented in Tables 2 and 3. In cross‐sectional studies, items 1 and 2 (Q1: Were the criteria for inclusion in the sample clearly defined?; Q2: Were the study subjects and the setting described in detail?) were considered not applicable, as these items were not informative for discriminating methodological quality within the included studies, given their shared focus on individuals with Down syndrome and the exclusion of known neuropsychiatric comorbidities, with limited variability across clinical, demographic, or contextual characteristics.

**TABLE 2:** Analysis of cross‐sectional studies.

Author/year	Q1	Q2	Q3	Q4	Q5	Q6	Q7	Q8	Quality
Rihtman et al. (2010)	No	Yes	Yes	Yes	No	No	Yes	Yes	**
Wuang and Su (2011)	Yes	No	Yes	Yes	Yes	No	Yes	Yes	**
Will et al. (2016)	Yes	Yes	Yes	Yes	Yes	No	Yes	Yes	**
Daunhauer et al. (2017)	Yes	Yes	Yes	Yes	No	Yes	Yes	Yes	**
Will et al. (2021)	Yes	Yes	Yes	Yes	Yes	Yes	Yes	Yes	***
Onnivello et al. (2022)	Yes	Yes	Yes	Yes	Yes	Yes	Yes	Yes	***

*Note:* Good quality ***; Fair quality **; Low quality *. **Q1‐** Were the criteria for inclusion in the sample clearly defined? **Q2‐** Were the study subjects and the setting described in detail? **Q3‐** Was the exposure measured in a valid and reliable way? **Q4‐** Were objective, standard criteria used for measurement of the condition? **Q5‐** Were confounding factors identified? **Q6‐** Were strategies to deal with confounding factors stated? **Q7‐** Were the outcomes measured in a valid and reliable way? **Q8‐** Was appropriate statistical analysis used?

**TABLE 3:** Analysis of longitudinal studies.

Author/year	Q1	Q2	Q3	Q4	Q5	Q6	Q7	Q8	Q9	Q10	Q11	Quality
Crombie and Gunn (1998)	Yes	Yes	Yes	Yes	Yes	No	Yes	Yes	Yes	Yes	Yes	***
Van Deusen et al. (2024)	Yes	Yes	Yes	No	Yes	Yes	Yes	Yes	Yes	Yes	Yes	**

*Note:* Good quality***; Fair quality **; Low quality *. **Q1‐** Were the two groups similar and recruited from the same population? **Q2‐** Were the exposures measured similarly to assign people to both exposed and unexposed groups? **Q3‐** Was the exposure measured in a valid and reliable way? **Q4‐** Were confounding factors identified? **Q5‐** Were strategies to deal with confounding factors stated? **Q6‐** Were the groups/participants free of the outcome at the start of the study (or at the moment of exposure)? **Q7‐** Were the outcomes measured in a valid and reliable way? **Q8‐** Was the follow up time reported and sufficient to be long enough for outcomes to occur? **Q9‐** Was follow up complete, and if not, were the reasons for loss to follow up described and explored? **Q10‐** Were strategies to address incomplete follow up utilised? **Q11‐** Was appropriate statistical analysis used?

Similarly, in longitudinal studies, items 1 (Q1: Were the two groups similar and recruited from the same population?) and 6 (Q6: Were the groups/participants free of the outcome at the start of the study?) were deemed not applicable, as the included designs did not involve comparisons between distinct groups or the assessment of outcome incidence over time but rather focused on continuous associations between cognitive variables and adaptive behaviour. Therefore, these items did not influence the overall assessment of methodological quality.

## 4 Discussion

Compiled evidence indicates a consistent correlation between IQ scores and different areas of adaptive behaviour, although there is a recurring perception that these scores vary over development, with reports of progressive decline (Rihtman et al. 2010), potentially related to early Alzheimer onset, which deserves to be better understood and treated. In Crombie and Gunn (1998), verbal cognition was strongly associated with the practical domain of adaptive behaviour, while nonverbal cognition showed a correlation of smaller magnitude. Besides the signs that deficits in attention, inhibition, working memory, and set‐shifting are early findings in individuals with Down Syndrome coursing with Alzheimer's dementia, how the executive functioning in childhood interacts with the early onset of Alzheimer in individuals with Down syndrome is still to be fully clarified (Grigorova et al. 2023).

Executive functions emerged as central predictors of adaptive behaviour in children with Down Syndrome. Executive functions encompass higher‐order control skills that allow individuals to focus on new or complex tasks when automatic processes are insufficient. Their main components, cognitive flexibility, inhibitory control and working memory, support higher‐level functions such as reasoning, planning, problem‐solving and organisation (Hocking et al. 2024). Within the cognitive domain, it was observed that these skills are indeed selectively impaired in individuals with Down syndrome (Loveall et al. 2017).

Working memory was the domain most consistently associated with adaptive behaviour, showing a substantial effect size, besides the varied methodological quality of the studies (Will et al. 2021; Daunhauer et al. 2017). Increasing evidence indicates that working memory is one of the most impaired cognitive skills in children with Down syndrome (Nacinovich et al. 2021). Increasing social demands in adolescence, which may impact social adaptation function even as the overall cognitive skills remain relatively stable (Nacinovich et al. 2021). Long‐term follow‐ups in this population had commonly shown a decline in adaptive behaviour after the age of 30, often progressing towards signs of early dementia (Carr and Collins 2018). The early Alzheimer's‐type neuropathology characteristic of may influence adaptive behaviour (Arvio and Luostarinen 2016). On the other hand, positive environmental factors and resilience may partially mitigate this decline (Boyle et al. 2025).

Inhibitory control is less investigated, but seems particularly robust in Van Deusen et al. (2024), remaining as a substantial predictor of daily living skills. Previously, there were inconsistent results about the inhibitory control association with adaptive behaviour (Hocking et al. 2024). Actually, there is data suggesting a highlight of the significant role of inhibitory control as an important predictor of successful adaptive behaviour in a fair‐quality longitudinal study.

Cognitive flexibility was also a significant predictor, but its role may be varied (Van Deusen et al. 2024). Both verbal and nonverbal cognition seem to predict adaptive behaviour. In this longitudinal study, verbal cognition primarily influenced communication, while nonverbal cognition impacted practical and social autonomy (Crombie and Gunn 1998). Recent findings confirm impairments in communication as a critical factor for adaptive behaviour performance (Nacinovich et al. 2021).

Higher‐order executive functions, such as planning, have been less studied than other executive components. Onnivello et al. (2022) conducted a methodologically rigorous study and identified moderate associations between planning difficulties and aspects of adaptive behaviour, particularly within the practical domain. In a study of children with Down syndrome, Lee et al. (2011) observed significantly poorer planning scores compared to typically developing peers. Amado et al. (2016) reported more pronounced deficits in social skills, belonging to the social domain of adaptive behaviour. Daunhauer et al. (2017) did not find evidence that planning was a significant predictor of adaptive behaviour. So, the available findings remain heterogeneous.

In addition to executive functions, visual organisation has also been identified as a vulnerable area in children with Down syndrome, although existing studies show significant methodological limitations. Wuang and Su (2011) found moderate effect sizes between visual skills and all domains of adaptive behaviour. Complementarily, Rihtman et al. (2010) reported that both integrated and isolated visuomotor skills showed moderate correlations with overall adaptive behaviour.

Attention was examined in a school setting in the study by Will et al. (2016). Attentional difficulties were negatively associated with practical adaptive behaviour, specifically the ability to engage in and to complete tasks. Jacola et al. (2014) reported that attention problems in adolescents with Down syndrome were associated with difficulties across multiple adaptive behaviours, including daily living activities, functional communication, adaptability and social skills.

In the current digital context, the adaptability of children with Down syndrome may face additional challenges that reflect the cognitive and adaptive behaviour deficits identified in this review. There is extensive exposure to technologies such as televisions, tablets, smartphones and computers, but cognitive and language difficulties may compromise the effective use of these resources (Morris et al. 2024; Feng et al. 2008; 2010). Moreover, phonological and speech articulation deficits affect performance in automatic speech recognition systems, limiting user autonomy (Cibrian et al. 2025). Even when visual and auditory aids enhance attention and engagement, motor barriers, such as difficulties with a mouse, keyboard, or voice commands, may continue to significantly restrict independent technology use (Morris et al. 2024).

Difficulties related to adaptive behaviour are highly frequent in Down syndrome, making it necessary to evaluate and to plan interventions for better functioning (Schalock et al. 2010). Recognising the critical link between executive functions and adaptive behaviour means that interventions for children with intellectual deficiency should not only focus on teaching specific adaptive behaviour but also on developing underlying executive function abilities. Cognitive limitations significantly influence the level of adaptive functioning, but the interventions considering the executive functioning might facilitate intervention planning, which should include task chaining, visual support and schedules, self‐monitoring strategies and role‐playing and social stories, as observed as necessary in each individual.

## 5 Limitations

This review has some limitations that should be considered. Although the studies employed standardised and validated instruments to assess adaptive behaviour, in most cases responses were provided by parents, which may not fully capture the child's performance across different contexts. Additionally, the small number of included studies and the predominance of cross‐sectional designs limit the breadth and strength of the evidence. There is a clear need for longitudinal studies that follow children from early childhood through to adolescence to establish a clear causal path and to confirm whether early EF deficits truly precede and cause later adaptive declines.

## 6 Conclusion

Cognitive skills in children with Down Syndrome, particularly executive functions, are directly related to performance in daily living activities and personal independence, which are central components of adaptive behaviour. This study contributes to the understanding of the strengths and limitations of the cognitive profile of children with Down Syndrome, as well as their impact on functional outcomes. Overall, adaptive behaviour is a multifaceted process, influenced not only by environmental factors, such as daily autonomy support provided by parents, but also by cognitive factors. In this context, relevant questions emerge for future research, including: what are the most effective ways to stimulate cognitive skills in children with Down Syndrome, and how can intervention programmes targeting executive functions be structured across different stages of child development?

## Author Contributions

**Fernanda Silva Pereira and Renata Maria Silva Santos:** conceptualization, methodology, investigation, writing – original draft preparation, writing – review and editing. **Luiz Humberto Souza Junior, Núbia Hadassa França Ferreira de Carvalho, Clara de Paula Gomes and Alice Martins Ferreira:** investigation, data curation. **Marco Aurélio Romano‐Silva:** supervision. **Leandro Fernandes Malloy‐Diniz:** supervision. **Débora Marques de Miranda:** validation, writing – original draft preparation and supervision.

## Funding

This project is funded by the government agencies Fundação de Coordenação de Aperfeiçoamento de Pessoal de Nível Superior (CAPES), Ministry of Science, Technology, and Innovation (MCTI), National Council for Scientific and Technological Development (CNPq 406935/2022‐0), the National Institute of Science and Technology‐INCT‐Neurotec‐R, and FAPEMIG.

## Conflicts of Interest

The authors declare no conflicts of interest.

## Supporting information


**Table S1:** Search Strategies.

## Data Availability

Data sharing not applicable to this article as no datasets were generated or analysed during the current study.
