# Supplementary material for: Cognitive Predictors of Adaptive Behaviour in Children With Down Syndrome: A Systematic Review
Source: J Appl Res Intellect Disabil. 2026 Feb 17;39(1):e70201. doi: 10.1111/jar.70201 (PMC12914088; doi:10.1111/jar.70201)
Supplement: Supplementary file 1 — Table S1: Search Strategies. [file JAR-39-e70201-s001.docx]

**Cognitive Predictors of Adaptive Behavior in Children with Down Syndrome: A Systematic Review**

**Supplementary Material 1**

**Table S1 - Search Strategies**

| **Database** | **Syntax** |
| --- | --- |
| PsycInfo | Title: “Children” *AND* Title: "Down syndrome" *OR* Title: "trisomy 21" *AND* Title: "cognitive function" *OR* Title: "executive function" *OR* Title: "memory" *OR* Title: "intellectual function" *AND* Title: "adaptive behavior" *OR* Title: "adaptive functioning" *OR* Title: "daily living skills" *AND* Age Group: Preschool Age (2-5 yrs) *AND* Age Group: School Age (6-12 yrs) |
| Pubmed | All Fields: (“Children”) AND ("Down syndrome" OR "trisomy 21") AND ("cognitive function" OR "executive function" OR "memory" OR "intellectual function") AND ("adaptive behavior" OR "adaptive functioning" OR "daily living skills" OR "social skills") |
| Eric | All Fields: (“Children”) AND ("Down syndrome" OR "trisomy 21") AND ("cognitive function" OR "executive function" OR "memory" OR "intellectual function") AND ("adaptive behavior" OR "adaptive functioning" OR "daily living skills" OR "social skills") |
| Web of Science | ALL=((“Children”) AND ("Down syndrome" OR "trisomy 21") AND ("cognitive function" OR "executive function" OR "memory" OR "intellectual function") AND ("adaptive behavior" OR "adaptive functioning" OR "daily living skills" OR "social skills")) |
| Scopus | ALL=((“Children”) AND ("Down syndrome" OR "trisomy 21") AND ("cognitive function" OR "executive function" OR "memory" OR "intellectual function") AND ("adaptive behavior" OR "adaptive functioning" OR "daily living skills" OR "social skills")) |
